# *Brucella* Antibodies in Alaskan True Seals and Eared Seals—Two Different Stories

**DOI:** 10.3389/fvets.2018.00008

**Published:** 2018-01-31

**Authors:** Ingebjørg H. Nymo, Rolf Rødven, Kimberlee Beckmen, Anett K. Larsen, Morten Tryland, Lori Quakenbush, Jacques Godfroid

**Affiliations:** ^1^Arctic and Marine Biology, UiT – The Arctic University of Norway, Tromsø, Norway; ^2^Bioscience, Fishery and Economy, UiT – The Arctic University of Norway, Tromsø, Norway; ^3^Division of Wildlife Conservation, Alaska Department of Fish and Game, Fairbanks, AK, United States

**Keywords:** harbor seal, ribbon seal, ringed seal, serology, spotted seal, Steller sea lion, Northern fur seal, disease

## Abstract

*Brucella pinnipedialis* was first isolated from true seals in 1994 and from eared seals in 2008. Although few pathological findings have been associated with infection in true seals, reproductive pathology including abortions, and the isolation of the zoonotic strain type 27 have been documented in eared seals. In this study, a *Brucella* enzyme-linked immunosorbent assay (ELISA) and the Rose Bengal test (RBT) were initially compared for 206 serum samples and a discrepancy between the tests was found. Following removal of lipids from the serum samples, ELISA results were unaltered while the agreement between the tests was improved, indicating that serum lipids affected the initial RBT outcome. For the remaining screening, we used ELISA to investigate the presence of *Brucella* antibodies in sera of 231 eared and 1,412 true seals from Alaskan waters sampled between 1975 and 2011. In eared seals, *Brucella* antibodies were found in two Steller sea lions (*Eumetopias jubatus*) (2%) and none of the 107 Northern fur seals (*Callorhinus ursinus*). The low seroprevalence in eared seals indicate a low level of exposure or lack of susceptibility to infection. Alternatively, mortality due to the *Brucella* infection may remove seropositive animals from the population. *Brucella* antibodies were detected in all true seal species investigated; harbor seals (*Phoca vitulina*) (25%), spotted seals (*Phoca largha*) (19%), ribbon seals (*Histriophoca fasciata*) (16%), and ringed seals (*Pusa hispida hispida*) (14%). There was a low seroprevalence among pups, a higher seroprevalence among juveniles, and a subsequent decreasing probability of seropositivity with age in harbor seals. Similar patterns were present for the other true seal species; however, solid conclusions could not be made due to sample size. This pattern is in accordance with previous reports on *B. pinnipedialis* infections in true seals and may suggest environmental exposure to *B. pinnipedialis* at the juvenile stage, with a following clearance of infection. Furthermore, analyses by region showed minor differences in the probability of being seropositive for harbor seals from different regions regardless of the local seal population trend, signifying that the *Brucella* infection may not cause significant mortality in these populations. In conclusion, the *Brucella* infection pattern is very different for eared and true seals.

## Introduction

Alaskan waters accommodate a number of pinniped species, both true seals (family *Phocidae*) including Eastern North Pacific harbor seals (*Phoca vitulina richardsii*), spotted seals (*Phoca largha*), ribbon seals (*Histriophoca fasciata*), Arctic ringed seals (*Pusa hispida hispida*), and bearded seals (*Erignathus barbatus*), as well as eared seals (family *Otariidae*) including Steller sea lions (*Eumetopias jubatus*) and Northern fur seals (*Callorhinus ursinus*) (1). Currently, bearded seals of the Bering Sea distinct population segment, and Steller sea lions in the western distinct population segment have been listed as threatened (“*likely to become an endangered species within the foreseeable future*”) under the Endangered Species Act (ESA). Although the Northern fur seal Pribilof Island stock has not been listed under the ESA, they have been deemed depleted (“*below its optimum sustainable population*”) under the Marine Mammal Protection Act (MMPA) (2). Listings for seals were based on their predicted negative responses to climate change, while for sea lions and fur seals it is due to population declines for unknown reasons. Furthermore, the International Union for Conservation of Nature and Natural Resources (IUCN) Red List of Threatened Species considers several of these species as “Data deficient” (“*inadequate information to make a direct, or indirect, assessment of its risk of extinction based on its distribution and/or population status”*) (3). Clearly there are concerns about how well these species and populations are able to adapt to future climate change scenarios where disease prevalence is predicted to increase as new species with novel pathogens appear to exploit warmer waters and longer open water seasons, and the host–pathogen balance may be altered (4). Thus, health and disease status of these animal populations are of prime importance for the purpose of management and conservation.

*Brucella* spp. was first reported in true seals in 1994 (5) and *Brucella pinnipedialis* has since been isolated from numerous true seal species (6). Persistence in macrophages—causing chronic infections—is the hallmark of brucellosis (7). However, *B. pinnipedialis* isolated from harbor (*Phoca vitulina vitulina*) and hooded seals (*Cystophora cristata*), both true seal species, did not multiply *in vitro* in human, murine or hooded seal macrophages, or in human and hooded seal epithelial cells (8–10) suggesting it may be a less virulent *Brucella* subspecies with a lower zoonotic potential. Furthermore, hooded and harbor seal brucellae are attenuated in the BALB/c mouse model (11, 12), in contrast to virulent pathogenic terrestrial brucellae, such as *Brucella suis*, which show a great ability to multiply and persist in this model (12). *Brucella* infections are further characterized by bacterial replication in the reproductive system of primary hosts, associated with pathology in the reproductive organs, causing abortion and sterility (7). Interestingly, although *B. pinnipedialis* has often been detected, pathology associated with it in true seals is virtually absent (6).

Previous studies on hooded seals from the east side of Greenland (13) and harbor seals from Alaska and the East coast of the USA (14, 15) have shown an age-dependent serological pattern, with a low probability of being seropositive for pups, a higher probability for yearlings, followed by a decreasing probability with age. This indicates that exposure occurs during the first year of life rather than *in utero* with a subsequent clearing of the infection (13). However, whether a similar age-dependent serological pattern is present in other Alaskan seal species and populations had not been documented.

In contrast to the situation in true seals, brucellae are rarely isolated or detected by PCR in eared seals, making it difficult to evaluate the presence or absence of *Brucella*-associated pathology in these species. However, it is worth noticing that the few cases reported in eared seals have been associated with reproductive pathology (16–18) and that transplacental transmission has been suggested (16). Furthermore, certain eared seal species are able to host infections with the zoonotic strain type (ST) 27 (16) and terrestrial brucellae (19), and could hence pose a zoonotic risk.

Serologic tests for detecting antibodies against a specific etiologic agent are the first screening tools for wildlife. The Rose Bengal test (RBT) is a simple and reliable test recommended by the World Organization for Animal Health (OIE) for the detection of *Brucella* antibodies. However, when using it in marine mammals, fat globules being wrongly identified as agglutinates may interfere with the interpretation of the results (20, 21). Serum lipids may be partly removed by chloroform cleanup (20) and has previously been shown to greatly improve the agreement between RBT and an enzyme-linked immunosorbent assay (ELISA) when detecting *Brucella* antibodies (21). The first objective of the present study was to compare the results of RBT and ELISA in a subset of serum samples before and after chloroform cleanup. Thereafter, the second objective was to use the best technique to determine the seroprevalence of *Brucella* antibodies adjusting by other potential covariates.

For the remaining screening, we used ELISA to investigate the seroprevalence of *Brucella* antibodies in a large number of harbor seals, spotted seals, ribbon seals, ringed seals, Steller sea lions, and Northern fur seals from Alaskan waters, sampled between 1975 and 2011. The aim of the present study was to analyze how the likelihood of seropositivity varied between species, sex, age, sampling year, and regions in order to draw further conclusions on whether a *Brucella* infection may be negatively impacting the health and population dynamics of these species. Knowledge about to what degree these species harbor the infection is also of importance as many of these species are subsistence harvested and hence may pose a zoonotic threat.

## Materials and Methods

### Sampling

**Figure 1:**
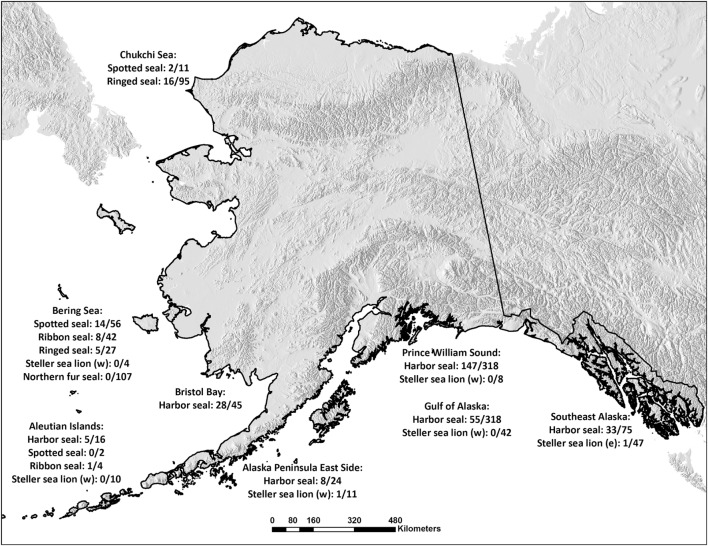
Number of *Brucella*-seropositive seals per species and sampling area. Serum samples obtained from Alaskan harbor seals, spotted seals, ribbon seals, ringed seals, Steller sea lions from the Western (w) and Eastern (e) distinct population segments, and Northern fur seals in the Chukchi and Bering Seas, in Bristol Bay, around the Aleutian Islands, on the Eastern side of the Alaska Peninsula, in Prince William Sound, in the Gulf of Alaska, on the coast of Southeast Alaska and in Argo Bay, Canada. The samples were investigated for the presence of *Brucella* antibodies and the numbers of positives per species and sampling spot are given.

Samples were collected (1975–2011) from Alaskan pinnipeds by biologists during live/capture release studies, scientific collections or Alaska native subsistence harvested animals and stored at −40 to −80°C at the Alaska Department of Fish and Game (ADF&G) in Fairbanks, Alaska until subsampled for this study (*n* = 1,643). Sample sizes and distribution by sex and age category (pups; <1 year, juveniles; 1–3 years, adults; >3 years) are depicted in Table 1. Age category was known for 1,420 seals (86%); 1,039 harbor seals (93%), 73 spotted seals (86%), 50 ribbon seals (91%), 75 ringed seals (50%), 46 Steller sea lions from the Western distinct population segment (61%), 31 Steller sea lions from the Eastern distinct population segment (65%), and 106 Northern fur seals (99%). Age by year was determined by assessing morphometric measurements (22), tooth annuli [e.g., (23)] or claw annuli [e.g., (24)] as validated for each species. Age by year was determined for 916 seals (56%): 599 harbor seals (53%, 0–30 years), 58 spotted seals (68%, 0–25 years), 49 ribbon seals (89%, 1–25 years), 75 ringed seals (50%, 0–16 years), 28 Steller sea lions from the Western distinct population segment (37%, 0 – 10 years), 15 Steller sea lions from the Eastern distinct population segment (31%, all pups), and 93 Northern fur seals (87%, all pups). The animals included in the study were from Alaskan waters (Figure 1) and seven ringed seals were from Argo Bay, Canada.

**Table 1:** Alaskan seals analyzed for *Brucella* antibodies.

Species	Pups (f/m/u)	Juveniles (f/m/u)	Adults (f/m/u)	Unknown (f/m/u)	Total (f/m/u)
Harbor seal	244 (120/123/1)	311 (154/156/1)	484 (239/242/3)	83 (32/43/8)	1,122 (545/564/13)
Spotted seal	12 (5/7/0)	34 (16/18/0)	27 (16/11/0)	12 (1/6/5)	85 (38/42/5)
Ribbon seal	0 (0/0/0)	21 (10/11/0)	29 (14/15/0)	5 (0/4/1)	55 (24/30/1)
Ringed seal	7 (3/4/0)	16 (6/10/0)	52 (20/31/1)	75 (25/40/10)	150 (54/85/11)
Steller sea lion (WDPS)	23 (9/14/0)	8 (6/2/0)	15 (13/2/0)	30 (9/12/9)	76 (37/30/9)
Steller sea lion (EDPS)	15 (8/7/0)	0 (0/0/0)	16 (16/0/0)	17 (0/0/17)	48 (24/7/17)
Northern fur seal	93 (60/32/1)	0 (0/0/0)	13 (13/0/0)	1 (0/0/1)	107 (73/32/2)
Total	394 (205/187/2)	390 (192/197/1)	636 (331/301/4)	223 (67/105/51)	1,643 (795/790/58)

*Age categories (pups; <1 year, juveniles; 1–3 years, adults; >3 years) and sex (f, females; m, males; u, unknown) for harbor seals (1975–2001), spotted seals (1978–2008), ribbon seals (1978–2003), ringed seals (1978–2011), Steller sea lions from the Western (1977–1996) and Eastern (1993–1995) distinct population segment, and Northern fur seals (1996–2000) investigated for Brucella antibodies in the present study*.

### Antibody Detection

Serum samples were analyzed for *Brucella* antibodies using a Protein A/G ELISA, as previously described (25). A subsample of sera was also tested using RBT (IDEXX Laboratories, Pourquier, Hoofddorp, the Netherlands) (20 ELISA-negatives/species, *n* = 140, and up to 20 ELISA-positives/species, *n* = 66). These sera (*n* = 206) were cleaned with chloroform to remove lipids (20, 21) and re-analyzed by ELISA (ELISA^chl^) and RBT (RBT^chl^).

### Statistical Analysis

Pairwise agreement among the serological tests before and after chloroform cleanup was assessed (Cohen’s kappa, κ) (26, 27). RBT and RBT^chl^ results were categorized as negative, positive, or impossible to interpret. ELISA and ELISA^chl^ results were categorized as negative or positive. The remaining statistical analyses were performed using ELISA results.

Differences in seroprevalence between sex and age groups were estimated using generalized linear models with a binomial error distribution and a logit link. To account for the possibility that age effects may differ between males and females, the interaction *age* × *sex* was included in the models. Influence of age was analyzed in two ways: first, age was treated as a categorical variable with three age categories: pups, juveniles, and adults. Second, for animals older than pups, we used age as a continuous variable. Each species was modeled separately instead of using “species” as a covariate to reduce the number of model parameters.

We limited the examination of how seropositivity varied spatially to harbor seals because this was the only species with a sufficient sample size from all sampling regions (Figure 1), with exception of the Aleutian Islands, which was excluded from the analysis. Region was modeled as a categorical variable. Age category and sex, as well as their interactions, were included as covariates in the full model. Not all regions, age categories, sex, and species, were sampled all years. Hence, we chose not to include sampling year as a covariate as preliminary analysis indicated that this could bias estimates, in particular for species with small sample sizes. However, a univariate analysis of trends in seroprevalence over time did not reveal any overall trend (logit regression, slope = 0.00, 95% CI = −0.02, 0.02).

The most parsimonious models where selected using Akaikes information criterion corrected for small sample sizes (AICc), using an all possible regression approach [library *MuMIn* in R (28)] on the sample size for all possible combinations (i.e., a fixed minimum sample size for all models ranked). Estimates are considered significant if their 95% confidence intervals do not include zero. All statistical analyses were performed in the program R, version 3.3.0 [R (29)].

## Results

Results from testing sera without chloroform cleanup (i.e., containing lipids), revealed moderate agreement between ELISA and RBT (κ: 0.66, SE: 0.04). Chloroform cleanup greatly improved agreement between the ELISA/ELISA^chl^ (identical results) and RBT^chl^ (κ: 0.90, SE: 0.03), while the agreement between RBT and RBT^chl^ was only moderate (κ: 0.68, SE: 0.04). Detailed information about how the chloroform cleanup affected the results are available in Table 2. The remaining results are ELISA results.

**Table 2:** Comparison of results before and after chloroform cleanup.

Tests		RBT			ELISA/ELISA^chl^	

	Results	Positive	Negative	Unknown	Positive	Negative
RBT^chl^	Positive	41	1	19	61	0
	Negative	4	128	7	3	136
	Unknown	0	2	4	2	4

ELISA/ELISA^chl^	Positive	42	2	22	66	0
	Negative	3	129	8	0	140

*Serum samples (*n* = 206) were analyzed for Brucella antibodies using a Protein A/G indirect enzyme-linked immunosorbent assay (ELISA) and the Rose Bengal test (RBT). The samples were thereafter subjected to a chloroform cleanup to remove lipids and re-analyzed by ELISA (ELISAchl) and RBT (RBTchl). RBT and RBTchl results were categorized as negative, positive, or impossible to interpret (unknown). ELISA and ELISAchl results were categorized as negative or positive. The results are pairwise compared in the table*.

*Brucella* antibodies were detected in 276/1,122 harbor seals (24.6%), 16/85 spotted seals (18.8%), 9/55 ribbon seals (16.4%), and 21/150 ringed seals (14.0%). *Brucella* antibodies were detected in 2/124 Steller sea lions, both of unknown age, one from the Western distinct population segment (1.3%) and one from the Eastern distinct population segment (2.1%). All 107 Northern fur seals were seronegative.

**Figure 2:**
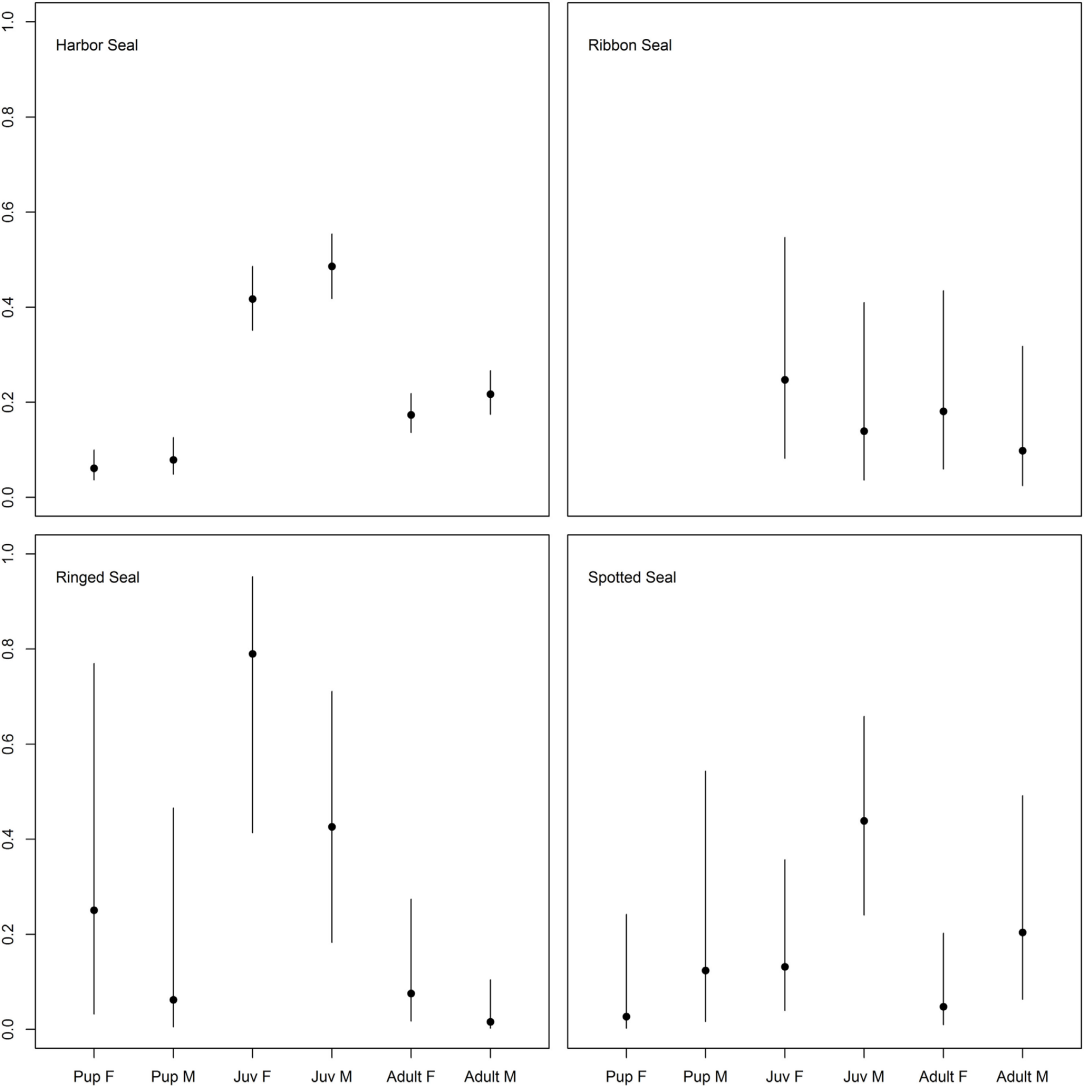
Differences in probability of being seropositive between age categories in true seals. Differences in probability of being *Brucella* spp. seropositive among age categories (pups < 1 year, juveniles 1–3 years, adults > 3 years) and sexes (males; M and females; F) for the four true seal species. Points indicate predicted mean, while error bars indicate 95% confidence intervals.

For harbor and ringed seals, juveniles had a higher probability of being seropositive than pups, and adults for harbor seals (45.3%, 95% CI = 40.0, 50.9 versus 7%, 95% CI = 4.2, 10.6 and 19.4%, 95% CI = 16.1, 23.1, and 56.3%, 95% CI = 32.3, 78.2 versus 14.3%, 95% CI = 0.9, 49.4 and 3.8%, 95% CI = 0.6, 11.4), though for the ringed seals the contrast to pups was not significant (Figure 2; Table 3; Table S1 in Supplementary Material). There was no such significant age category pattern evident for spotted or ribbon seals in the best approximating models (Figure 2; Table 3). While lower ranked models for both species included age category (δAICc < 2, Table S1 in Supplementary Material), these estimates did not significantly differ (95% CI for contrasts of pups and adults versus juveniles for spotted seal: [−4.71, 0.19] and [−2.75, 0.27], and for adults versus juveniles for ribbon seal: [−1.17, 1.95], all logit-transformed).

**Table 3:** Parameter estimates (logit-transformed) for best approximating models, with age is included as a categorical predictor.

Species	Predictor	Estimate	SE	*Z*-value	95% CI
Harbor seal	Intercept	−1.56	0.14	−10.93	(−1.85, −1.28)
*n* = 1,034	Age category—juveniles	1.23	0.16	7.55	(0.91, 1.55)
	Age category—pups	−1.18	0.28	−4.25	(−1.75, −0.66)
	Sex—males	0.28	0.15	1.79	(−0.02, 0.58)
Ribbon seal	Intercept	−1.63	0.36	−4.48	(−2.41, −0.97)
*n* = 55					
Ringed seal	Intercept	−2.51	0.77	−3.27	(−4.38, −1.23)
*n* = 74	Age category—juveniles	3.83	0.99	3.85	(2.10, 6.14)
	Age category—pups	1.41	1.34	1.06	(−1.79, 3.99)
	Sex—males	−1.62	0.90	−1.80	(−3.67, 0.02)
Spotted seal	Intercept	−2.14	0.53	−4.05	(−3.35, −1.22)
*n* = 80	Sex—males	1.22	0.63	1.95	(−0.06, 2.58)

*For age category, adult is set as reference level, while for sex, female is. SE is standard error, CI is confidence interval, and *n* is sample size used for parameter estimation (see Materials and Methods for details)*.

**Figure 3:**
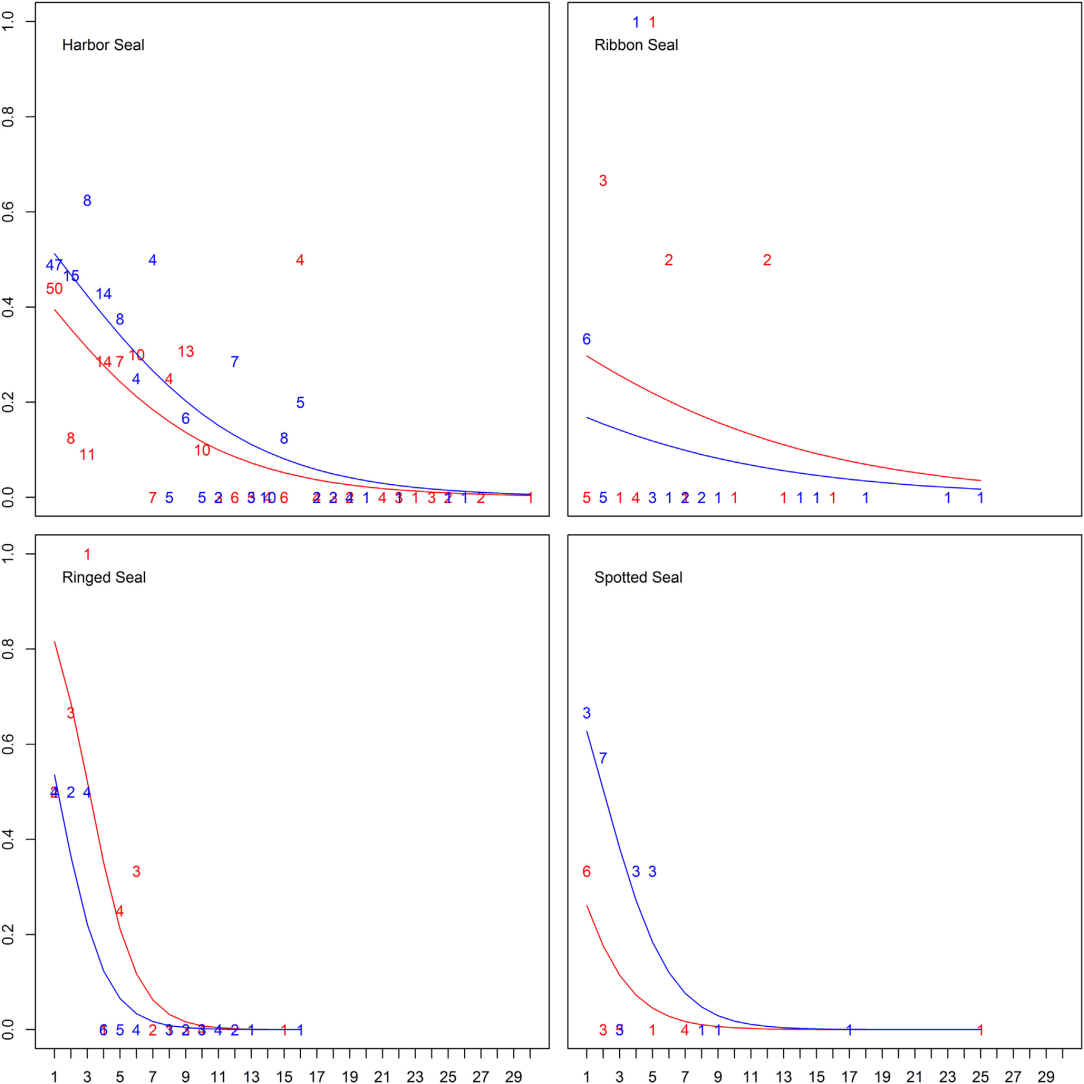
Differences in probability of being seropositive between ages in years for true seals. Differences in probability of being *Brucella* spp. seropositive among four true seal species at different age and sex (males in blue, females in red). Lines show the predicted probabilities, while numbers indicate empirical frequencies for different ages.

When splitting up age categories into age by year, there was an overall significant decreasing probability of being seropositive with age from the age of 1 year for all true seals except ribbon seals (Figure 3; Table 4; Table S2 in Supplementary Material). While seropositive animals were absent among individuals older than 5 years in spotted seals and 6 years in ringed seals, they were present among harbor seals until the age of 16, though at a very low prevalence for older ages (Figure 3).

**Table 4:** Parameter estimates (logit-transformed) best approximating models, when age is included as a continuous predictor (for animal equal to, or older than 1 year).

Species	Predictor	Estimate	SE	*Z*-value	95% CI
Harbor seal	Intercept	−0.25	0.23	−1.10	(−0.70, 0.19)
*n* = 351	Age	−0.18	0.03	−5.84	(−0.24, −0.12)
	Sex—males	0.47	0.26	1.82	(−0.04, 0.99)
Ribbon seal	Intercept	−1.63	0.39	−4.23	(−2.47, −0.93)
*n* = 49					
Ringed seal	Intercept	2.18	1.06	2.06	(0.28, 4.57)
*n* = 67	Age	−0.70	0.22	−3.17	(−1.21, −0.33)
	Sex—males	−1.35	0.86	−1.56	(−3.18, 0.29)
Spotted seal	Intercept	−0.54	0.86	−0.63	(−2.33, 1.13)
*n* = 46	Age	−0.50	0.27	−1.86	(−1.12, −0.08)
	Sex—males	1.56	0.82	1.93	(0.04, 3.34)

*For sex category, female is set as reference level. SE is standard error, while CI is confidence interval, and *n* is sample size used for parameter estimation (see Materials and Methods for details)*.

There was an inconsistent pattern for difference in probability of seropositivity between sexes (Tables S1 and S2 in Supplementary Material). Spotted seal males had a significant and harbor seal males a near significant higher probability of being seropositive, while the pattern was the opposite for ringed seals (Figures 2 and 3; Tables 3 and 4). Like ringed seals, ribbon seal showed higher probability for seropositivity among females, though not significant [95% CI = [−0.66, 2.54] and [−0.78, 2.44], for lower ranked age category (δAICc = 1.4) and age models (δAICc = 1.2), respectively].

**Figure 4:**
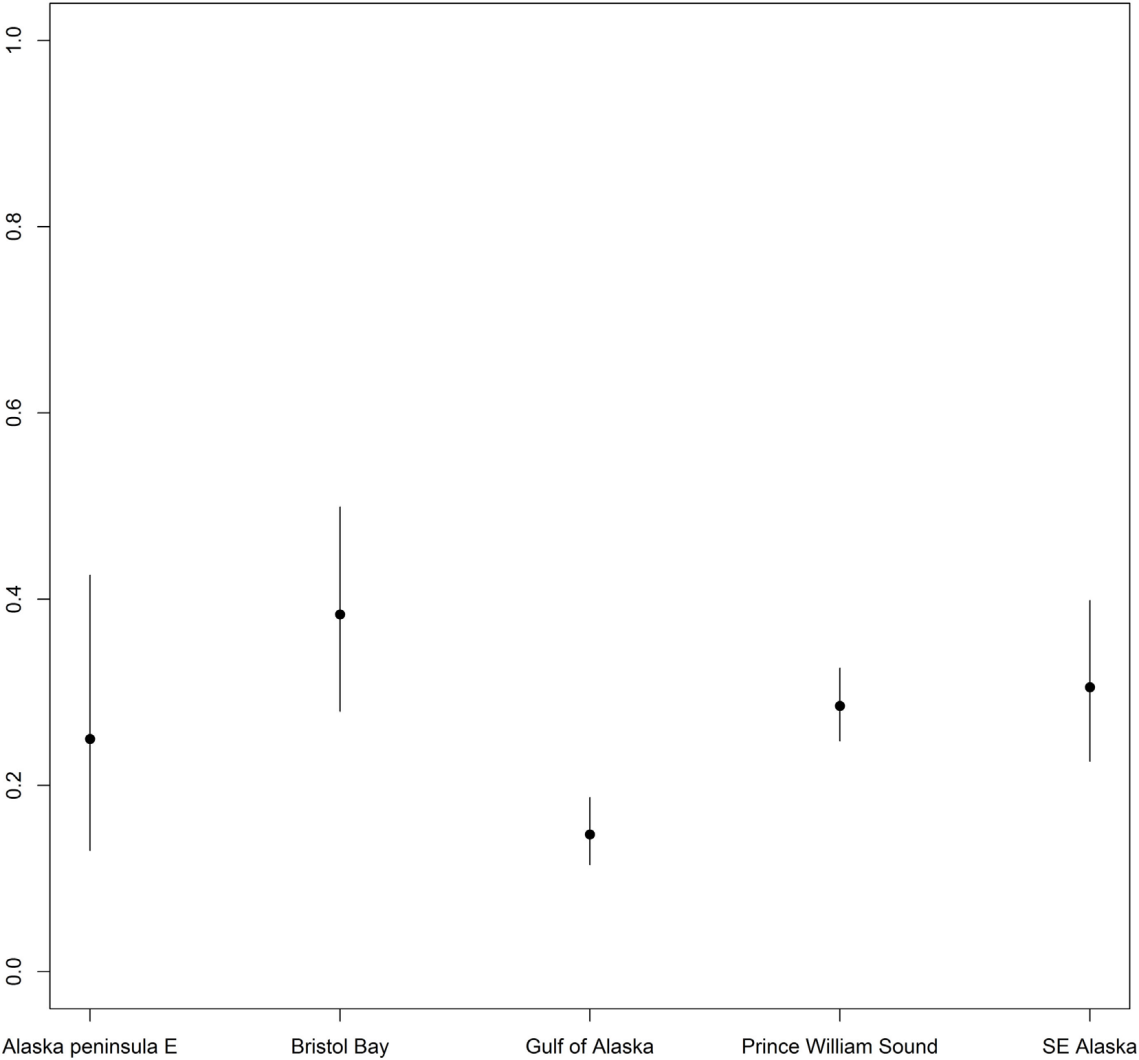
Differences in probability of being seropositive between harbor seals sampled at various regions. Differences in probability of being *Brucella* spp. seropositive between harbor seals sampled at various regions in Alaska. Points indicate predicted mean for all age groups, while error bars indicate 95% confidence intervals.

Differences were found in seropositivity among regions (δAIC = 9.2 to the best model not including region, controlled for age category and sex), mainly due to a significant lower probability of being seropositive for harbor seals in the Gulf of Alaska (Figures 1 and 4).

## Discussion

Past evaluations of *Brucella* infection status in marine mammals have likely been inaccurate if the results were based solely on agglutination tests. RBT is a much used, simple, cheap, and reliable test recommended by the OIE (30); however, the results may be influenced by the presence of hemolysis and globules of fat (21), often present in marine mammal sera. For marine mammals, it is necessary to run a combination of tests to determine if discrepancies are found between RBT and other serological tests. A chloroform cleanup may be required, followed by repetition of the tests, as shown in the present study where the agreement between ELISA and RBT was moderate, while after chloroform cleanup, the coherence between ELISA and RBT^chl^ was greatly improved. The improved agreement between the tests and the reduced number of non-interpretable RBT-results following chloroform cleanup indicate that the original serum quality likely contained lipids that reduced the accuracy of the initial RBT-outcome.

Even with serological techniques that are less sensitive to poor serum quality and lipemic serum (e.g., ELISA), positive serological results presume exposure (31), as serological cross-reactions and false positives bias (i.e., inflate) the results. Several potential agents cross-reacting with *Brucella* are identified (32), however, little is known about their presence in wildlife. Therefore, the gold standard in brucellosis diagnostics is bacterial isolation or detection of *Brucella* spp. specific DNA-fragments. Ideally serological tests should be used to screen sera and seropositives should be further tested by bacterial isolation and PCR to address *Brucella* status among Alaskan pinnipeds in the future.

An age-dependent serological pattern, with a low probability of being seropositive for pups, a higher probability for yearlings, followed by a decreasing probability with age, previously identified in hooded (13) and harbor seals (14, 15) was identified in harbor seals in the present study, and similar patterns were present also for the other true seal species, however, solid conclusions could not be made due to sample size. Harbor and ringed seal juveniles had a higher probability of being seropositive than pups and adults. Likewise, when analyzing juveniles and adults by exact ages, there was a falling probability of seropositivity with age for harbor, spotted and ringed seals. Seropositive animals were lacking among individuals older than 5 years in spotted seals and 6 years in ringed seals, but were present among harbor seals until the age of 16, though at a very low prevalence. Almost all seropositive ribbon seals were between 1 and 6 years, though one was 12 years. The lack of significant results for ribbon seals, although the same trend was present, is likely due to the small number of animals sampled, and the lack of pups among the sampled animals. These results suggest that the investigated true seal species may be clearing the infection with increasing age.

Seals have an endotheliochorial placenta where 5–10% of the maternal antibodies are transferred to the fetus *in utero* and the rest are transferred through the colostrum. The immunity transmitted is determined by the level of systemic immunity in the mother (33). The low numbers of seropositive harbor, spotted, and ringed seal pups in this study indicates that these (1) have not received maternal antibodies against *Brucella* and (2) are not exposed to brucellae and hence have not mounted an antibody response yet. The low seroprevalence among pups is consistent with our finding that the majority of the females had no detectable levels of *Brucella* antibodies by the time they reached sexual maturity.

*Brucella* spp. in terrestrial animals causes reproductive pathology and may be transmitted during breeding and lactation or by crossing the placenta from mother to offspring (34). However, reproductive pathology is not associated with *B. pinnipedialis* infections in true seals and vertical transmission of *B. pinnipedialis* has never been described in a true seal species (6). The limited number of serologically and bacteriologically positive true seals, of reproductive age, in previous studies (13, 15, 35, 36), and the herein presented age-dependent serological patterns, further indicates that maternal transmission is unlikely as females have become seronegative for *Brucella* by the time they reach sexual maturity. Furthermore, the mean probability of being seropositive increased from pups to juveniles in previous studies (13–15) as well as in this study, suggesting that exposure to *B. pinnipedialis* is primarily during the post-weaning period and during the first few years of life, and is not transmitted *in utero* or to neonates.

Although underlying reason for this age-related serological pattern is unknown, it may be related to changes in diet. Stable isotopes and mercury biomarkers have indicated that in general, adult harbor and ringed seals feed at a higher trophic level than pups (37). Stable isotope analysis has shown that ribbon and spotted seals also feed at increasing trophic levels with age (38). Hence, there may be a general shift in diet composition toward higher trophic levels with increasing age, which coincides with the age at which seropositive juveniles start to appear, indicating a possible reservoir of *B. pinnipedialis* in one or more lower trophic level prey species.

Analysis by region showed a decrease in the probability of being seropositive for harbor seals in the Gulf of Alaska compared to the other regions. At Nanvak Bay, the largest haul-out in northern Bristol Bay, harbor seals declined in abundance between 1975 and 1990, but have increased since (2). Samples from harbor seals were from the time period 1975 to 2001, however, as different sites were sampled different years, we chose not to include year as a covariate in the statistical analysis. Still, univariate analysis of trends in seroprevalence over time did not reveal any overall trend suggesting that the *Brucella* infection in the harbor seal population in Bristol Bay may not contribute to higher mortality rates.

Considering the lack of impact on the harbor seal population trends, the age-dependent serological and bacteriological patterns (13–15, 35, 36), the lack of *Brucella*-associated pathology in true seals (6) and the lack of multiplication in established *in vitro* (8–10) and *in vivo* models (11, 12), it is possible that true seals may not be the primary hosts of *B. pinnipedialis*, but rather a spillover host. *B. pinnipedialis* has been isolated from lungworms in seals (35) and a recent experimental infection showed that a *B. pinnipedialis* hooded seal strain survived in Atlantic cod (*Gadus morhua*) (39). Moreover, a novel *Brucella* strain has been isolated from a fish, a bluespotted ribbontail ray (*Taeniura lymma*) (40). In addition, *Brucella microti* has been isolated from soil (41) and novel brucellae strains have been isolated from frogs (42–44), indicating an extended ecological niche of brucellae. Further investigation of marine sources for exposure to *B. pinnipedialis* should be performed in order to further reveal the epizootiology of *Brucella* infection in true seals.

The infection pattern in eared seals seems to be very different from that found in true seals. We detected *Brucella* antibodies in only two Steller sea lions, and none of the Northern fur seals. These findings are consistent with the low number of *Brucella* isolates obtained from eared seals in other studies; four bacteriology positive California sea lion placentas (16, 17), of which two showed signs of inflammation and multifocal acute necrosis (17). Additionally, transplacental transmission of brucellae in California sea lions has been indicated when brucellae strains belonging to the zoonotic ST27 were detected by PCR in three placentas and multiple fetal tissues in parallel (16). Terrestrial brucellae of unknown origin have also been detected by PCR in blood and milk from two apparently clinically healthy wild California sea lions, and marine mammal brucellae were detected in blood and milk from one animal (19). *B. pinnipedialis* has also been detected by PCR in six Northern fur seal placentas, of which one had severe placentitis (18), and in one Northern fur seal spleen with no pathology associated (45). The low number of isolates and PCR-positive cases obtained from eared seals make drawing any conclusions regarding the presence or absence of pathology in these species difficult, however, it is worth noticing that the few cases reported have often been associated with pathology in the reproductive organs (16–18) and that transplacental transmission has been suggested (16). The low seroprevalences detected in eared seals of all ages in the present study suggests a low level of exposure due to possibly a different diet or a greater resistance toward the infection. Considering the reports of pathology in eared seals, morbidity and/or mortality due to infection is also possible. Further studies, including samples suited for bacterial isolation and/or PCR and from a higher number of individuals from different age groups, are needed to determine to what degree the infection is a threat to the Alaskan eared seal populations.

Certain eared seal species are able to host infections with the zoonotic ST27 (16) and terrestrial brucellae (19). There have been three cases of naturally acquired infections in humans with ST27, none of which had been in contact with marine mammals; however, they had been at the coast, eaten raw shellfish (46) or been in contact with raw fish bait (47). Further studies on both marine mammals and other species from the Arctic marine ecosystem are warranted in order to address this important issue, especially as marine mammals and other marine species are used for human consumption. Whether the zoonotic ST27 is present in Alaskan waters is currently unknown and warrants further investigation; however given the ample opportunities for transfer from marine mammals to humans, it appears that if ST27 were present more cases would be known.

In conclusion, the *Brucella* serological pattern is very different for true and eared seals. The infection in true seals seems to be relatively common, yet shown in the present study to be transient and decreasing with increasing age for harbor seals, becoming virtually absent at the age of sexually maturity. Similar patterns were present also for the other true seal species; however, firm conclusions could not be made due to sample size. This suggests that true seals may not be the primary hosts of *B. pinnipedialis*, but rather a spillover host susceptible to infection from other sources in the marine environment. In eared seals, we detected only two seropositive animals which could be explained by a low level of exposure or lack of susceptibility to infection; however, it could also be explained by high susceptibility to *Brucella* infection with mortality removing infected animals from the population. Comparison of true and eared seal *Brucella* isolates with established bacteriological and molecular methods (6) could provide new information about their potential differences and similarities. Furthermore, the pathogenicity of isolates should be compared to already characterized terrestrial *Brucella* strains in established *in vitro* cell (7) and *in vivo* mouse (12) *Brucella* models.

## Ethics Statement

This study was carried out in accordance with the recommendations of four Institutional Animal Care and Use protocols approved by two separate committees. These protocols were approved by the Alaska Department of Fish and Game’s Institutional Animal Care and Use Committee (protocols #06-16, 0921, #03-0014, 09-08, and #2010-13R) and by the University of Alaska Fairbanks’ committee (protocol #98-23).

## Author Contributions

Acquisition of samples: LQ and KB. Design of study: IN, KB, and JG. Laboratory testing: IN. Statistical analysis: RR. Interpretation of data: IN and RR. Drafting the work or revising it critically for important intellectual content: IN, RR, AL, MT, LQ, KB, and JG. All authors have read and approved the final manuscript.

## Conflict of Interest Statement

The authors declare that the research was conducted in the absence of any commercial or financial relationships that could be construed as a potential conflict of interest.
