# Supplementary material for: Brucella Antibodies in Alaskan True Seals and Eared Seals—Two Different Stories
Source: Front Vet Sci. 2018 Jan 31;5:8. doi: 10.3389/fvets.2018.00008 (PMC5797734; doi:10.3389/fvets.2018.00008)
Supplement: Supplementary file 1 [file Table_1.DOCX]

**Supplementary table S1**

Model selection table for models including age as a categorical predictor. AICc is the Akaike information criterion corrected for small sample sizes value, δAICc show differences compared to the most parsimonious model, while *n* is sample size used for model selection (se material and method section for details).

| **Species** | **Predictors** | **AICc** | **δAICc** |
| --- | --- | --- | --- |
| Harbor seal | β_0_ + age.cat + sex | 1030.1 | 0 |
| *n* = 1034 | β_0_ + age.cat | 1031.3 | 1.2 |
|  | β_0_ + age.cat + sex + age.cat x sex | 1032.6 | 2.5 |
|  | β_0_ + sex | 1147.3 | 117.3 |
|  | β_0_ | 1148.1 | 118.1 |
|  |  |  |  |
| Ribbon seal | β_0_ | 46.1 | 0 |
| *n* = 50 | β_0_ + sex | 47.4 | 1.4 |
|  | β_0_ + age.cat | 48.0 | 1.9 |
|  | β_0_ + age.cat + sex | 49.4 | 3.4 |
|  | β_0_ + age.cat + sex + age.cat x sex | 51.2 | 5.2 |
|  |  |  |  |
| Ringed seal | β_0_ + age.cat + sex | 49.4 | 0 |
| *n* = 74 | β_0_ + age.cat | 50.9 | 1.5 |
|  | β_0_ + age.cat + sex + age.cat x sex | 51.6 | 2.2 |
|  | β_0_ + sex | 67.6 | 18.2 |
|  | β_0_ | 67.7 | 18.3 |
|  |  |  |  |
| Spotted seal | β_0_ + sex | 69.3 | 0 |
| *n* = 73 | β_0_ + age.cat + sex | 69.4 | 0.1 |
|  | β_0_ + age.cat | 73.3 | 4.0 |
|  | β_0_ | 73.4 | 4.1 |
|  | β_0_ + age.cat + sex + age.cat x sex | 73.7 | 4.4 |
