# Supplementary material for: Brucella Antibodies in Alaskan True Seals and Eared Seals—Two Different Stories
Source: Front Vet Sci. 2018 Jan 31;5:8. doi: 10.3389/fvets.2018.00008 (PMC5797734; doi:10.3389/fvets.2018.00008)
Supplement: Supplementary file 2 [file Table_2.DOCX]

**Supplementary table S2**

Model selection table for models including age as a continuous predictor for individuals older than pups. AICc is the Aikaikes Information Criterion corrected for small sample sizes value, δAICc show differences compared to the most parsimonious model, while *n* is sample size used for model selection (se material and method section for details).

| **Species** | **Predictors** | **AICc** | **δAICc** |
| --- | --- | --- | --- |
| Harbor seal | β_0_ + age + sex | 355.8 | 0 |
| *n* = 351 | β_0_ + age | 357.1 | 1.3 |
|  | β_0_ + age + sex + age x sex | 357.7 | 1.8 |
|  | β_0_ + sex | 405.4 | 49.5 |
|  | β_0_ | 407.9 | 52.1 |
|  |  |  |  |
| Ribbon seal | β_0_ | 45.7 | 0 |
| *n* = 49 | β_0_ + age | 46.4 | 0.7 |
|  | β_0_ + sex | 46.9 | 1.2 |
|  | β_0_ + age + sex | 47.8 | 2.1 |
|  | β_0_ + age + sex + age x sex | 48.3 | 2.6 |
|  |  |  |  |
| Ringed seal | β_0_ + age + sex | 44.1 | 0 |
| *n* = 67 | β_0_ + age | 44.5 | 0.4 |
|  | β_0_ + age + sex + age x sex | 45.5 | 1.4 |
|  | β_0_ | 61.9 | 17.8 |
|  | β_0_ + sex | 62.7 | 18.6 |
|  |  |  |  |
| Spotted seal | β_0_ + age + sex | 47.2 | 0 |
| *n* = 46 | β_0_ + age | 48.9 | 1.8 |
|  | β_0_ + age + sex + age x sex | 49.6 | 2.4 |
|  | β_0_ + sex | 51.2 | 4.0 |
|  | β_0_ | 52.7 | 5.5 |
